# CD151 promotes Colorectal Cancer progression by a crosstalk involving CEACAM6, LGR5 and Wnt signaling via TGFβ1

**DOI:** 10.7150/ijbs.53657

**Published:** 2021-02-17

**Authors:** Tao Yang, Huibing Wang, Meng Li, Linqi Yang, Yu Han, Chao Liu, Baowen Zhang, Mingfa Wu, Gang Wang, Zhenya Zhang, Wenqi Zhang, Jianming Huang, Huaxing Zhang, Ting Cao, Pingping Chen, Wei Zhang

**Affiliations:** 1 Center for Medical Research and Innovation, Shanghai Pudong Hospital, Fudan University Pudong Medical Center, Shanghai, 201399, China.; 2 Department of Pharmacology, Hebei University of Chinese Medicine, Shijiazhuang, Hebei, 050200, China.; 3 Department of Pharmacy, Children's Hospital of Hebei Province, Shijiazhuang, Hebei, 050000, China.; 4 Department of Laboratory Animal Science, Hebei Key Lab of Hebei Laboratory Animal Science, Hebei Medical University, Shijiazhuang, Hebei, 050017, China.; 5 Hebei Collaboration Innovation Center for Cell Signaling, Key Laboratory of Molecular and Cellular Biology of Ministry of Education, Hebei Key Laboratory of Moleculor and Cellular Biology, College of Life Sciences, Hebei Normal University, Shijiazhuang, Hebei, 050024, China.; 6 Department of Gastrointestinal Surgery, Dingzhou City People's Hospital, Dingzhou, Hebei, 073000, China.; 7 Department of Third General Surgery, Cangzhou City People's Hospital, Cangzhou, Hebei, 061000, China.; 8 Department of Second General Surgery, Hebei Medical University Fourth hospital, Shijiazhuang, Hebei, 050011, China.; 9 College of Basic Medicine, Hebei Medical University, Shijiazhuang, Hebei, 500017, China.; 10 School of Basic Medical Sciences, Hebei Medical University, Shijiazhuang 050017, Hebei, China.

**Keywords:** CD151, TGFβ1, LGR5, CEACAM6, Wnt signaling, colorectal cancer

## Abstract

CD151 impacts various signaling pathways in different cancers, and promotes colorectal cancer (CRC) cell malignancy by yet undefined mechanisms. This study aimed to comprehensively assess CD151's function in CRC. CD151 levels were significantly higher in CRC tissues and cells compared with controls in the tissue microarray. Cell viability, migration and invasion were suppressed by CD151 downregulation in CRC cells. Consistently, mouse xenografts were inhibited by CD151 silencing. RNA-seq revealed that multiple genes were significantly altered by CD151 knockdown in cultured CRC cells and xenografts. Particularly, transforming growth factor β1 (TGFβ1), carcinoembryonic antigen-related cell adhesion molecule 6 (CEACAM6) and leucine-rich repeat-containing G-protein coupled receptor 5 (LGR5) alongside CD151 were downregulated both *in vitro* and *in vivo*. Co-immunoprecipitation and mass spectrometry results were validated by qRT-PCR and immunoblot. Moreover, pull-down assay and immunofluorescence confirmed the associations of TGFβ1, CEACAM6 and LGR5 with CD151. This study demonstrated CEACAM6, LGR5 and Wnt pathway suppression by CD151 silencing might occur through TGFβ1 regulation, offering a comprehensive view of CD151's roles in colorectal carcinogenesis. Our findings provide an insight into the CD151-involved signaling network in CRC oncogenesis, which could be utilized to design novel targeted therapies against CD151-based signaling in treatment for CRC.

## Introduction

Colorectal cancer (CRC) is one of the most malignant cancers around the world, causing death mostly by locoregional relapse and metastasis. CRC represents a growing health concern worldwide, especially in developing countries as the “Western” lifestyle is increasingly adopted 1. Surgery and chemotherapy are commonly administered to CRC patients 2. Currently, designing effective targeted therapies for cancer remains an important clinical challenge.

CD151 was reported to be associated with cancer cell's aggressive cell proliferation and invasiveness in various malignancies such as lung, colon, breast, liver and prostate cancers, through interactions with laminin-binding α3β1, α6β1, and α6β4 integrins 3-5. CD151 expression is considered a poor prognostic factor in solid tumors such as colon cancer, breast cancer, gastric cancer, and esophageal squamous cell carcinoma. Indeed, a number of studies have shown that significant overexpression of CD151 is related to tumor cell motility, adhesion, proliferation and metastasis 6-8. In addition, high expression of CD151 in all stages and subtypes of epithelial ovarian cancer was demonstrated by RNA-seq analysis 9. However, several reports have indicated that CD151 expression depends on the stage of tumor progression 10-12. In colorectal cancer patients, CD151 expression is high in early disease stage, but decreases with increasing invasion and metastasis caused by hypoxia 13,14.

CD151 is involved in many signaling pathways. For example, its overexpression upregulates matrix metallopeptidase 9 (MMP9) through the PI3K/Akt/GSK-3/Snail pathway in hepatocellular carcinoma (HCC) 15-17. In addition, CD151-α3β1 integrin complexes regulate ovarian tumor growth by repressing Slug-mediated epithelial-mesenchymal transition (EMT) and Wnt signaling 18. Furthermore, CD151 induces cancer metastasis by regulating transforming growth factor (TGF) β1 in breast cancer 19. Moreover, CD151 is considered a major contributor to Wnt oncogene-driven tumorigenesis, impacting breast cancer malignancy 20. However, the mechanism of CD151's effects in colon cancer progression remains largely unknown.

Carcinoembryonic antigen-related cell adhesion molecule (CEACAM) 6 overexpression was reported in colonic hyperplastic polyps, adenomas, and adenocarcinomas, and was shown to have strong associations with colorectal cancer and increased invasiveness in liver CRC metastasis 10. CEACAM6 is also regulated by human epidermal growth factor receptor 2 (HER2) through the TGFβ pathway 11. Meanwhile, leucine rich repeat-containing G protein-coupled receptor 5 (LGR5), a colorectal cancer stem cell marker, is correlated with TGFβ and Wnt signaling, which could increase cell proliferation, survival, and metastasis in colon cancer 12,21,22.

Because complex genetic and non-genetic factors are involved in tumorigenesis, manipulating CD151 expression may be fundamental to understanding its key role in CRC tumorigenesis. Therefore, the present study is aimed to assess the functional role of CD151 in CRC and to explore the mechanism underlying its effects. Our findings provide an insight into the CD151-involved signaling networkin oncogenesis of CRC, which could be utilized to design novel targeted therapies against CD151-based signaling during the treatment for CRC.

## Materials and Methods

### Collection of carcinoma tissue samples

Colorectal cancer tissue and blood samples were collected from patients enrolled in the Second Department of General Surgery, Hebei Medical University Fourth Hospital (China). All tissue samples were immediately processed after surgical removal. Diagnosis and grading were histologically confirmed by two experienced pathologists. The study was conducted in accordance with the Declaration of Helsinki (as revised in 2013). This study was approved by the Ethical Committee of Hebei Medical University Fourth Hospital and Shanghai Pudong Hospital. Written consents were obtained from all patients.

### Other samples analyzed

This study assessed 598 clinically excised colon adenocarcinoma and rectum adenocarcinoma specimens, from the TCGA database (https://tcga-data.nci.nih.gov/tcga/).

### Tissue microarray (TMA) construction and immunohistochemistry (IHC)

A total of 80 clinical specimens were collected in the Second Department of General Surgery, Hebei Medical University Forth Hospital in China. TMA construction has been described in detail as reported 23. Briefly, after paraffin-embedding, 2-mm diameter tissue cylinders were punched from representative tissue areas. For IHC, 4-μm sections were deparaffinized in xylene and successively rehydrated with 85% and 75% ethanol. Endogenous peroxidase activity was blocked with 3% hydrogen peroxide, antigen retrieval was performed by microwaving, and nonspecific binding was blocked via incubation with 10% normal rabbit serum (since primary antibodies were originated from the goat) or 3% bovine serum albumin (BSA; Solarbio, Beijing, China) at room temperature for 30 min. TMA slides were incubated with anti-CD151 antibody (1:50; Antibody Revolution, Boston, MA, USA) overnight at 4 °C. Diaminobenzidine (DAB; Wuhan Servicebio Technology Co., Ltd., Wuhan, China) reagent was used to detect positive cells with brown-yellow nuclei.

### Cell culture

Human colon cancer HT29 (HTB-38, American Type Culture Collection (ATCC), Manassas, VA, USA) and HCT116 (CCL-247, ATCC) cell lines were cultured in McCoy's 5A medium (GIBCO, Carlsbad, CA, USA) supplemented with 10% fetal bovine serum and penicillin (100 U/mL)/streptomycin (100 mg/mL) at 37 °C in a moisture-saturated atmosphere containing 5% CO_2_.

### DNA transfection

The lentiviral vector pLV[Exp]-Puro-U6>hCD151[shRNA]-CMV>Luciferase with sh-CD151 (CD151 knockdown) and HT29-NC or HCT116-NC (lentiviral vector alone) as a negative control were synthesized by Fitgene Biotech (Guangzhou, China). HT29 and HCT116 cells were seeded in 24-well plates at 50% confluence on the day before transfection. For viral infection, titrated viral stocks were suitably diluted in blank medium to obtain the expected multiplicity of infection (MOI), and added to HT29 or HCT116 cell monolayers in wells containing polybrene at 5 μg/ml. The medium with the lentivirus was replaced with complete medium after 6 h. CD151 silencing in HT29 or HCT116 cells was screened by puromycin (2 μg/ml) treatment after 48 h. In addition, qRT-PCR was carried out to evaluate CD151 expression in cells.

### MTT assay

HT29-NC, HCT116-NC, sh-CD151 HT29 and sh-CD151 HCT116 cells were cultured in 96-well plates for 24, 48 and 72 h at 37 °C, respectively. The 3-(4,5-dimethylthiazol-2-yl)-2,5-diphenyltetrasolium bromide (MTT) assay was conducted for cell proliferation assay. Briefly, after treatment, 20 μL of 5 mg/mL MTT solution was added to 200 μL of medium for 4 h of incubation. Optical density (OD) was obtained on a spectrophotometer at 570 nm.

### Wound-healing assay

HT29-NC, HCT116-NC, sh-CD151 HT29 and sh-CD151 HCT116 cells (1.0×10^5^) were seeded in 24-well cell culture plates, respectively, and grown overnight to a confluent monolayer. A pipette tip (10 μL) was used to generate a scratch on the cell culture, followed by two washes with phosphate-buffered saline (PBS). After culture for 48 h in McCoy's 5A medium supplemented with 2% serum, the migration of cells was evaluated by comparing the area difference between 0 and 48 h via a Motic digital medical image analysis system (Leica DM2500, Heidelberg, Germany).

### Transwell chamber invasion assay

HT29, HCT116, HT29-NC, HCT116-NC, sh-CD151 HT29, and sh-CD151 HCT116 cells (2×10^4^) were added to transwell chambers (Corning, NY, USA), respectively. Serum was added to the bottom wells of the transwell system to induce cell invasion. After 48 h, the cells that migrated through the membrane were stained with 0.5% methylrosaniline chloride solution and counted in 5 random high-power fields. The data were represented as percent of the average number of cells per field under a light microscope.

### Xenograft colon tumor mouse models

Male BALB/C nude mice (6-8 week old; 20-25g) were purchased from Beijing Vital River Laboratory Animal Technology Co., Ltd. (China) and housed in a laminar flow cabinet under specific pathogen-free conditions. The animal experiments were approved by the Institutional Animal Care and Use Committee of Hebei Medical University (approval ID: SYXK2018-005).

Logarithmically growing cells were harvested after trypsinization, washed twice with McCoy's 5A medium and resuspended in sterile PBS. Cell suspensions (0.2 mL) adjusted to 2×10^6^/ml were injected subcutaneously into the flank right foreleg of each nude mouse. BALB/c nude mice were randomly divided into four groups, including the HT29-NC (n=6), HT29 sh-CD151 (n=6), HCT116-NC (n=7) and HCT116 sh-CD151 (n=7) groups.Meanwhile, mice were treated with firefly luciferin (150 mg/kg, Sciencelight; Shanghai, China) by intraperitoneal injection for bioluminescence image (BLI) analysis. Then, the nude mice were anesthetized with isoflurane 10 min post luciferin injection. BLIs were collected on an IVIS Spectrum CT *in vivo* Imaging System (PerkinElmer Inc., Waltham, MA, USA). Mouse body weights and tumor volumes were recorded every two days, with daily observations. The tumor volumes were calculated as V = (a×b^2^)/2 (cm^3^), where a and b are the tumor's long and short diameters, respectively, to construct growth curves. The tumor tissues were excised and weighed after 14 days. Each tumor sample was immediately frozen in liquid nitrogen and stored at -80 °C.

### RNA sequencing

To obtain clear insights into the mechanism of CD151 in colon cancer in cultured cells and xenografts, RNA sequencing (RNA-seq) was performed to determine its impact on the transcriptomic profile (n=3). Total RNA was isolated with TRIzol Reagent (Invitrogen, Carlsbad, CA, USA) from triplicate samples of HT29-NC and HT29 sh-CD151 cells, as well as mouse xenograft HT29-NC and HT29 sh-CD151 groups. RNA concentrations were between 23.8 and 41 ng/μl. RIN values were ~9.4 for RNA integrity as assessed on an Agilent Bioanalyzer 2100 (Agilent Technologies, Palo Alto, CA, USA). Paired-end libraries were synthesized with VAHTS Stranded mRNA-seq Library Prep Kit for Illumina® (Vazyme Biotech, Nanjing, China). Illumina sequencing libraries were constructed according to a modified strand-specific RNA-seq protocol 24. Library construction and sequencing were performed by Shanghai Biotechnology Corporation (China). Significantly expressed genes (SEGs) were selected with False Discovery Rate (FDR) < 0.05 and a fold-change ≥2. Enrichment analysis of differentially expressed genes (DEGs) was conducted to identify enriched gene ontology (GO) terms (http://www.geneontology.org/), as well as signal transduction and biochemical pathways in the Kyoto Encyclopedia of Genes and Genomes (KEGG) database 25.

### Co-immunoprecipitation (CoIP)

To determine proteins with potential interactions with CD151, CoIP was performed to assess whole cell extracts (WCEs) from HT29 cells. HT29 cells were lysed in lysis buffer (25 mM Tris, 150 mM NaCl, 1mM EDTA, 1% NP-40, 5% glycerol; pH 7.4). Total CD151 protein concentrations were measured by the BCA assay. CoIP was carried out with CoIP Kit (Pierce Co-immunoprecipitation kit; ThermoFisher Scientific; Waltham, MA, USA) according to the manufacturer's instructions. Briefly, HT29 cells were washed with PBS, lysed with cold immunoprecipitation lysate buffer, and centrifuged. Next, the cleared cell lysates were immunoprecipitated with anti-CD151 antibody or control normal mouse IgG (SC-2025) plus protein A Sepharose overnight at 4 °C. CD151 and its interacting proteins were precipitated with anti-CD151 antibody. SDS-polyacrylamide gel electrophoresis (PAGE) was performed, and the target proteins were extracted from the bands. The samples were analyzed by Mass Spectrometry, and CD151 interacting proteins were identified in a protein database.

### Mass Spectrometry

Selected protein bands were in-gel digested by trypsin as previously described 26,27. The extracted peptides were dissolved in 10 μl 0.1% formic acid. The peptide samples were loaded onto a C18 reverse phase column (100 μm 150 mm, ThermoFisher Scientific) coupled online to a QE-XF mass spectrometer (ThermoFisher Scientific) at a flow rate of 350 nl/min. Peptides were eluted with a gradient from 100% of A (0.1% formic acid) to 45% of B (0.1% formic acid and 95% acetonitrile) for 60 min.

### Real time fluorescence quantitative reverse transcription-polymerase chain reaction (qRT-PCR)

Total RNA from the abovementioned cells and tissues were isolated with ISOGEN reagent (Nippon Gene Co. Ltd., Kokyo, Japan) according to the manufacturer's instructions. RNA concentration and purity were assessed by spectrophotometry at 260 nm. A total of 1 µg of total RNA was converted into cDNA with Revert Aid first strand cDNA synthesis kit (ThermoFisher Scientific). The primers used are shown in **Table 1**. Real time fluorescence qRT-PCR was performed on a Real-Time PCR system (BIOER Co. Ltd., Kokyo, Japan) in reaction mixtures (20 μl) containing cDNA, primer pairs, and platinum SYBR Green QpcrSuperMix-UDG (Invitrogen) 28.

### Western blot

The cells and tissues were washed three times with PBS, lysed for 30 min on ice and centrifuged at 10,000×g at 4 °C for 5 min. Protein contents in cell lysates were quantified on a ND-1000 Spectrophotometer (NanoDrop, ThermoFisher Scientific). Equal amounts of total protein were separated by 10% SDS-PAGE and transferred onto PVDF membranes (Millipore, Billerica, MA, USA). Immunoblotting with primary antibodies was performed overnight at 4 °C after blocking with 5% milk for 2 h. The primary antibodies were raised against CD151 (1:200; Antibody Revolution), LGR5 (1:800; R&D Systems, Minneapolis, MN, USA), Wnt3a (1:500; R&D Systems), β-catenin (1:800; BD Biosciences, Bedford, MA, USA), TGFβ1 (1:1000; Abcam, Cambridge, MA, USA), Wnt5a (1:500; Affinity Biosciences, Changzhou, China), CEACAM6 (1:500; Affinity Biosciences), and β-actin (1:10000; Affinity Biosciences). Next, goat anti-rabbit (1:5000; Millipore) or goat anti-mouse (1:5000; Millipore) secondary antibodies were incubated for 2 h. Images were captured, and protein band intensities were analyzed with the Odyssey software (Odyssey Software, Denver, CO, USA).

### Immunofluorescence

CD151 and LGR5 expression levels were analyzed with confocal laser scanning microscopy to observe CD151 and LGR5 localizations in HCT116 cancer cells. HCT116 cells cultured on glass coverslips were formalin-fixed for 30 min, and cells were washed 3 times with PBS for 5 min. Triton X-100 was used to permeabilize the HCT116 cell membrane. After incubation with BSA for 30 min, the cells were simultaneously treated overnight at 4 °C with primary antibodies against CD151 (1:50) and LGR5 (1:50) followed by incubation with Alexa Fluor 488-labeled Goat Anti-Mouse IgG and Cy3-labeled Goat Anti-Rabbit IgG(H+L) (Wuhan Servicebio Technology Co., Ltd., Wuhan, China) secondary antibodies. Finally, 4',6-diamidino-2-phenylindole (DAPI) staining for 10 min was performed after PBS washes for thrice. Anti-fluorescence quenching sealant mounting was then carried out before observation with confocal microscopy.

### Plasmid construction and GST Pull-down assay

With CD151, LGR5, CEACAM6 and TGFβ1 plasmids uesd as templates, 1 μl of respective CD151, LGR5, CEACAM6 and TGFβ1 primers, and 10 μl prime STAR Max (2X) were mixed for PCR at 96 °C for 240 s, 96 °C for 20 s, 60 °C for 30 s, 72 °C for 90 s, and 70 °C for 180s. LGR5, CEACAM6 and TGFβ1 complementary DNAs were cloned into the BamHI/XhoI sites of pGEX4T-1; CD151 complementary DNA was cloned into the BamHI/XhoI sites of pET-28a (+). The coding sequences of LGR5, CEACAM6, TGFβ1 and CD151, were inserted into the glutathione S-transferase GST-tag pGEX4T-1 and His-tag pET-28a (+) expression vectors, respectively. After PCR amplification, recombinant pGEX4T-1-LGR5, pGEX4T-1-CEACAM6, pGEX4T-1-TGFβ1 and pET-28a (+)-CD151 were transformed into E. coli strain BL21. After verification by sequencing, the insert was released from LGR5 and CD151, and separately cloned into the prokaryotic GST-tag and His-tag fusion expression vectors. GST pull-down primers were shown in **Table 2**. The pGEX4T-1-LGR5, pGEX4T-1-CEACAM6, pGEX4T-1-TGFβ1 and pET-28a(+)-CD151 constructs were transformed into E. coli strain BL21, respectively. The transformed bacteria were grown onto LB medium (Invitrogen) with the OD 600 of the medium adjusted to 0.6, and induced with 0.1 mM isopropyl-1-thio-β-D-galactopyranoside (IPTG) at 20 °C for 3 h. SDS-PAGE revealed GST-LGR5 and His-CD151 in cell supernatants. GST-LGR5, GST-CEACAM6, GST-TGFβ1 and GST containing lysates were incubated with GST resin at 4 °C for 1 h, respectively. Each GST resin was incubated with His-tagged CD151 containing lysate overnight at 4 °C. After centrifugation at 4 °C for 3 min and 3 washes with lysis buffer, the protein complexes were eluted with 20 mM new reducing glutathione for 10 min. The pull-down complexes and bacterial lysate (input) were detected with anti-GST (1:6000; Sigma, St. Louis, MO, USA), anti-His (1:1000; Sigma), and rabbit IgG as secondary antibody (1:8000; Millipore) by Western blot.

### Statistical analysis

Data are mean ± standard deviation (SD) from the indicated number of independently performed experiments. Statistical significance (*P*<0.05) was assessed by one-way analysis of variance (ANOVA) followed by the S-N-K test. Log-rank (Mantel-Cox) survival analysis was performed to assess the association of patient prognosis with CD151 expression on basis of the TCGA database.

## Results

### CD151 is upregulated in CRC tissue samples and associated with reduced patient survival

**Figure 1:**
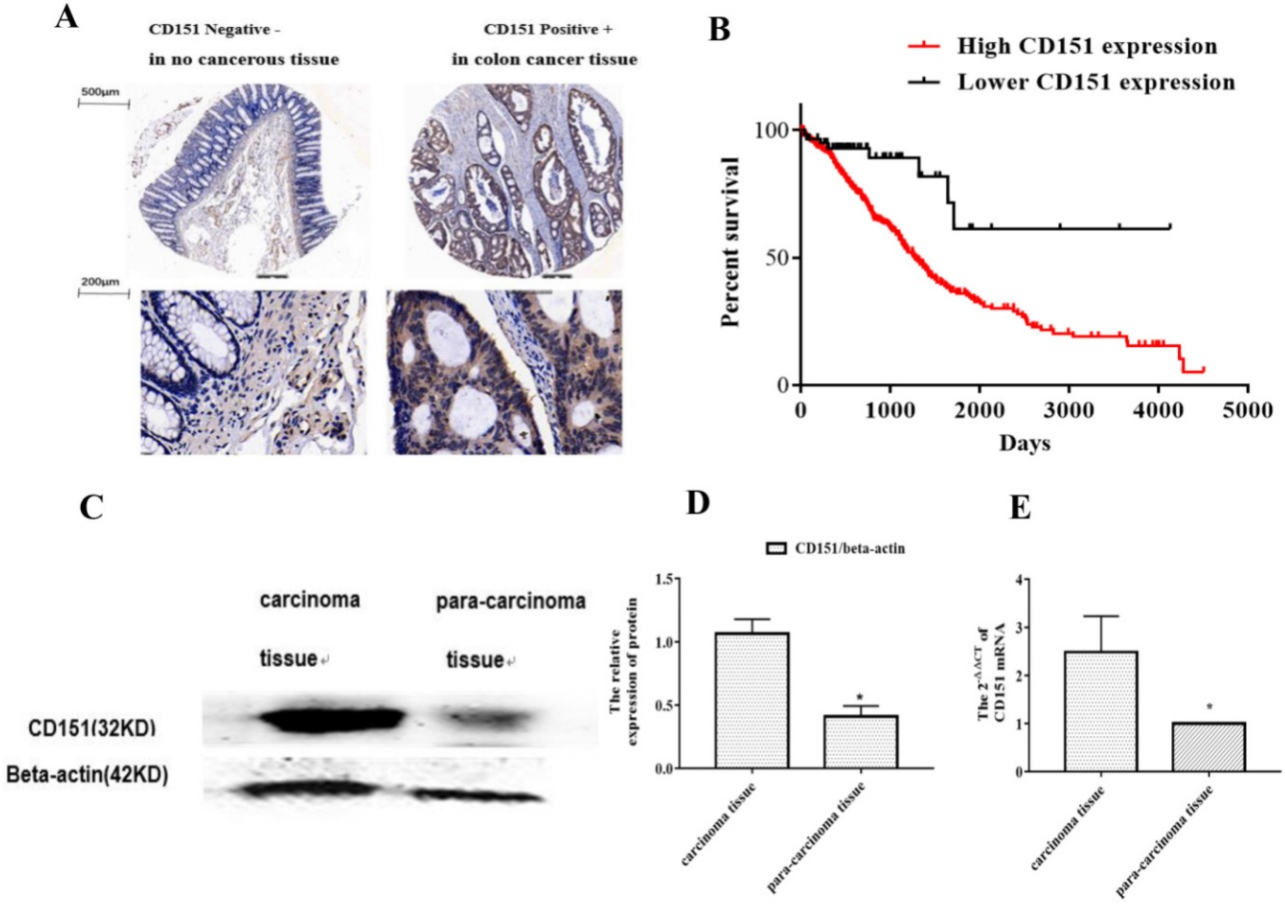
** CD151 is upregulated in colon cancer tissues. A.** Representative micrographs showing CD151 expression in colon cancer and paired non-cancerous tissue specimens as assessed by immunohistochemistry in tissue microarray (n=80). **B.** Log-rank (Mantel-Cox) survival analysis of CRC patients and CD151 expression on basis of the TCGA database indicated a negative correlation between CD151 expression and the survival rate of CRC patients (n=598; *P*=0.006, Chi-square=11.77). **C and D.** CD151 protein was upgraded in colon cancer tissues compared with paired non-cancerous samples as determined by Western blot (n=5). **E.** CD151 mRNA levels were increased in colon cancer specimens compared with paired non-cancerous tissues as assessed by qRT-PCR (n=32; *P<0.05).

To investigate whether CD151 protein expression is altered in CRC, a TMA slide was generated with CRC and para-carcinoma tissue samples from 80 patients. IHC was performed to evaluate CD151 expression and analyze its associations with clinicopathologic features. As shows in **Figure 1A**, epithelial structure in CRC was irregular, with significant variations in cell nucleus size and shape. The CD151 protein was highly expressed in CRC tissues. There was a negative correlation between CD151 expression levels and the survival rate of CRC patients. Further analysis showed a statistically significant association of CD151 expression with survival in all validation data sets (*P*=0.006, Chi-square=11.77). High CD151 expression (n=539) resulted in lower overall survival compared with the low CD151 expression group (n=59) in CRC patients (**Figure 1B**). CD151 expression was significantly increased in 5 colon cancer tissue samples compared with paired non-cancerous samples as assessed by Western blot (*P*<0.05, **Figure 1C and D**). In agreement, CD151 mRNA levels were increased in colon cancer tissue specimens compared with paired non-cancerous ones as determined by qRT-PCR (n=32, *P*<0.05, **Figure 1E**). The clinicopathological characteristics and CD151 expression statuses assessed by IHC in 80 CRC patients are summarized in **Table 3**. Two sided Fisher's exact analysis indicated a significant correlation between CD151 expression and distant metastasis (pM0 *vs* pM1; *P*=0.001). No significant associations between CD151 expression and other clinicopathologic variables were found.

### CD151 inhibits proliferation, migration and invasion of colon cancer cells

**Figure 2:**
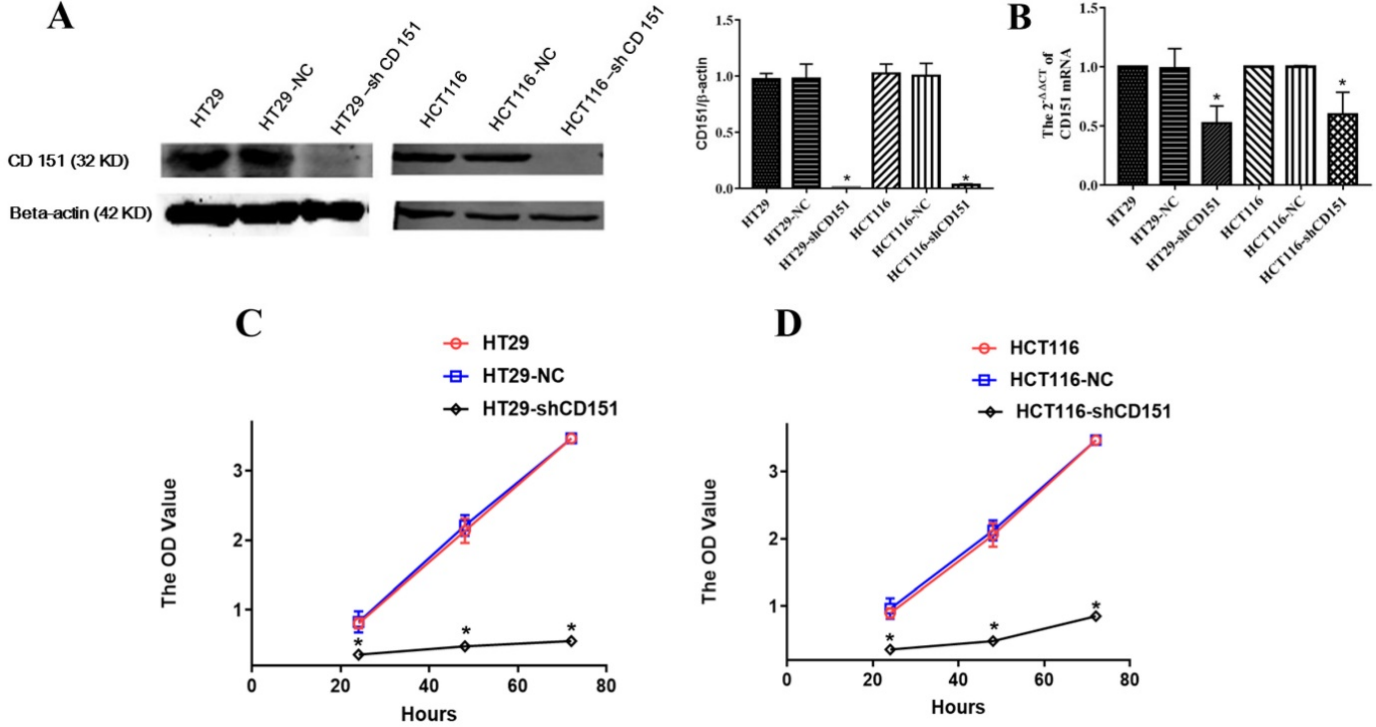
** CD151 knockdown inhibits the proliferation of HT29 and HCT116 cells. A and B.** Western blot and qRT-PCR confirmed that sh-CD151 significantly decreased CD151 protein and mRNA levels in HT29 and HCT116 cells. **C and D.** The MTT assay showed that sh-CD151 treatment inhibited cell proliferation of HT29 and HCT116 cells at 24, 48, 72 h, respectively. *P<0.05 compared with the negative control (HT29-NC or HCT116-NC) groups.

**Figure 3:**
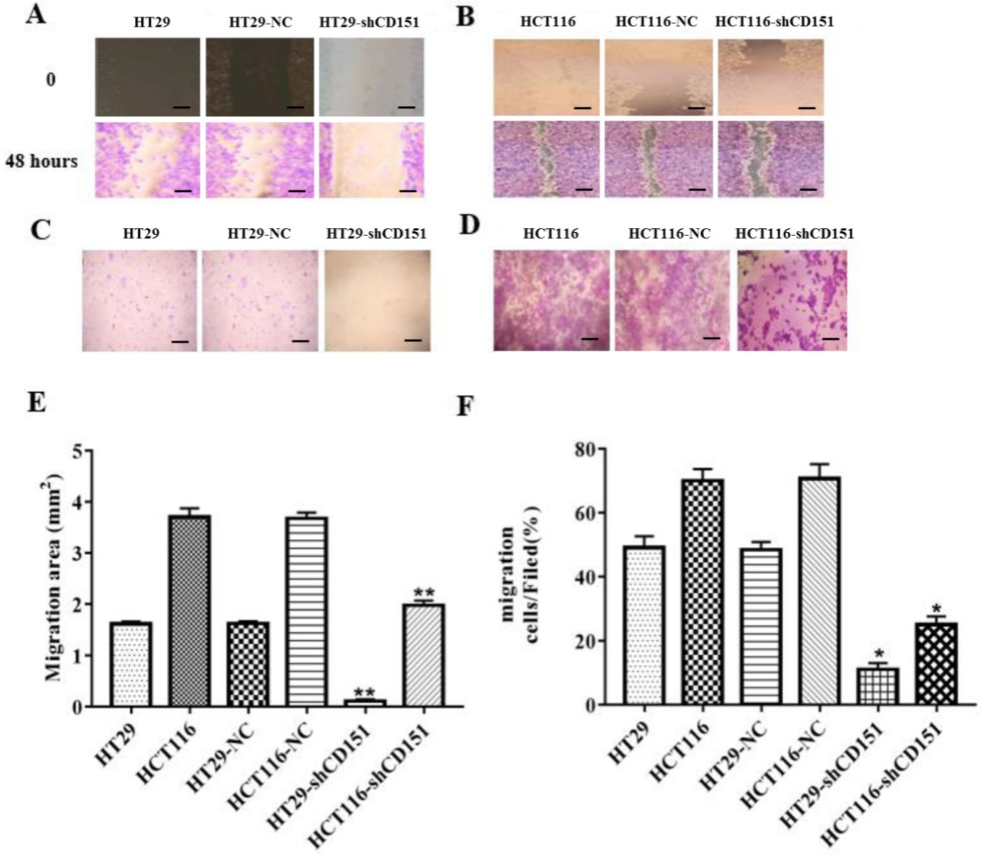
** Knockdown of CD151 inhibits migration and invasion of HT29 and HCT116 cells. A, B and E.** Migrative activities were suppressed in sh-CD151 HT29 and HCT116 cells as determined by the wound healing assay. **C, D and F.** Cell invasion abilities were inhibited in the transwell assay for both sh-CD151 HT29 and sh-CD151 HCT116 cells. Data are represented as mean ± SD derived from at least three independent experiments. * *P* < 0.05, **P < 0.01 compared with the negative control (HT29-NC or HCT116-NC) groups. Scale bars, 50 µm for A, B; 20 µm for C, D.

As shows in **Figure 2A and B**, CD151 was knocked down in HT29 and HCT116 cells, as examined by Western blot and qRT-PCR. To verify whether CD151 is required for colon cancer progression, we investigated cell proliferation, migration and invasion in sh-CD151 HT29 and HCT116 cells. As shown in **Figure 2C and D**, the sh-CD151 groups in both cell lines showed decreased cell viability at 24-72 h (all *P*<0.05). In wound healing assay, the cells migrated significantly less after CD151 silencing (**Figure 3A, B and E**; *P*<0.05). In agreement, the transwell assay indicated that cell migration and invasion were inhibited upon CD151 silencing (**Figure 3C, D and F**), in both HT29 and HCT116 cells (**Figure 3A-F**).

### CD151 knockdown suppresses colon cancer growth *in vivo*

**Figure 4:**
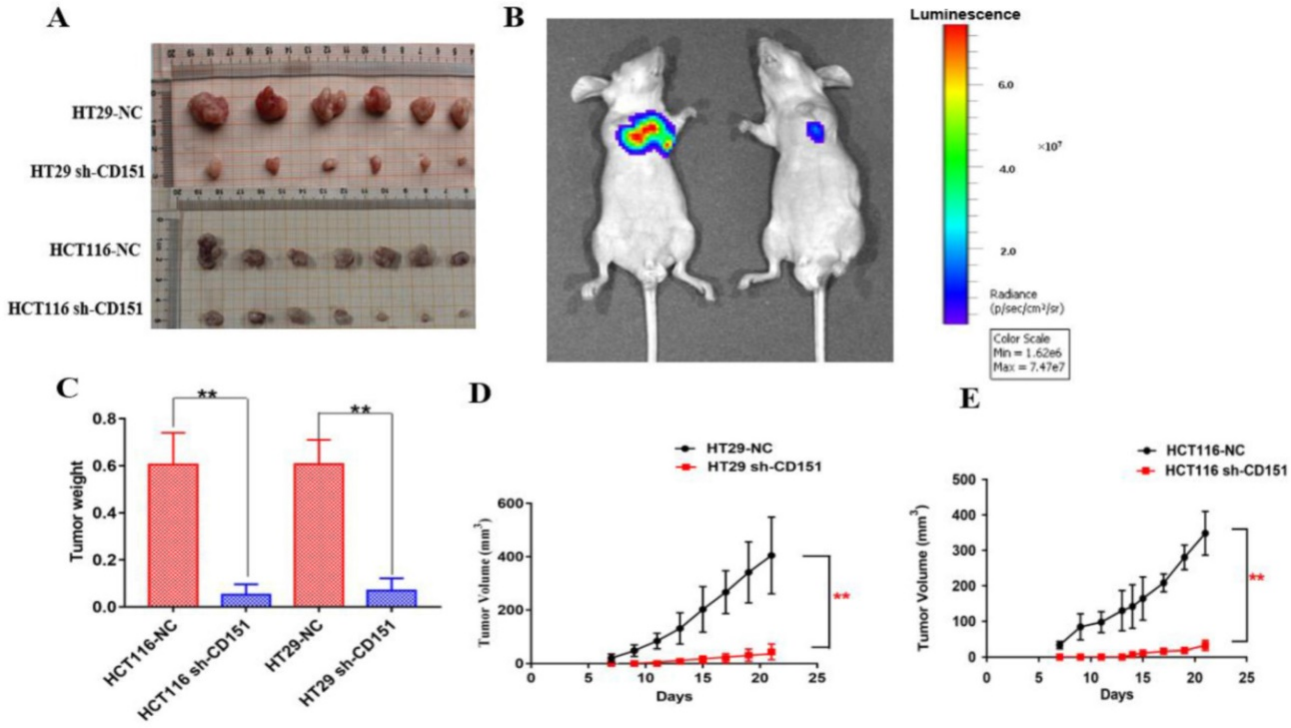
** Effects of CD151 knockdown on HT29- and HCT116-derived xenograft tumor growth in nude mice. A.** CD151 knockdown via sh-CD151 in HT29 and HCT116 cells decreased the growth rate of xenograft tumors in nude mice, respectively. **B.** Representative bioluminescent images of subcutaneous xenograft tumors in the control (left) and experimental groups derived from sh-CD151 HCT116 cell implantation (right). **C.** Detection of xenograft tumor weights in the control and experimental groups derived from sh-CD151 HCT116 or sh-CD151 HT29 cell implantation. **D and E.** Dynamic measurement of xenograft tumor volumes in the control and experimental groups derived from sh-CD151 HT29 (D) or sh-CD151 HCT116 (E) cell implantation. **P < 0.01.

To further investigate the role of CD151 in tumorigenesis, we evaluated the effect of CD151 knockdown on HT29 and HCT116 cell-related nude mouse xenografts. As shown in **Figure 4A, C-E**, tumor growth was significantly slower in the sh-CD151 groups compared with the control groups, with reduced tumor volumes and weights. The control group of HCT116 cells showed time-dependently increased luminescence, which was markedly reduced by CD151 silence. As shown in **Figure 4B**, luminescence decreased in the HCT116 sh-CD151 group indicating that tumor progression was repressed by reducing CD151 expression.

### CD151 knockdown alters multiple cancer-associated biological processes in colon cancer

**Figure 5:**
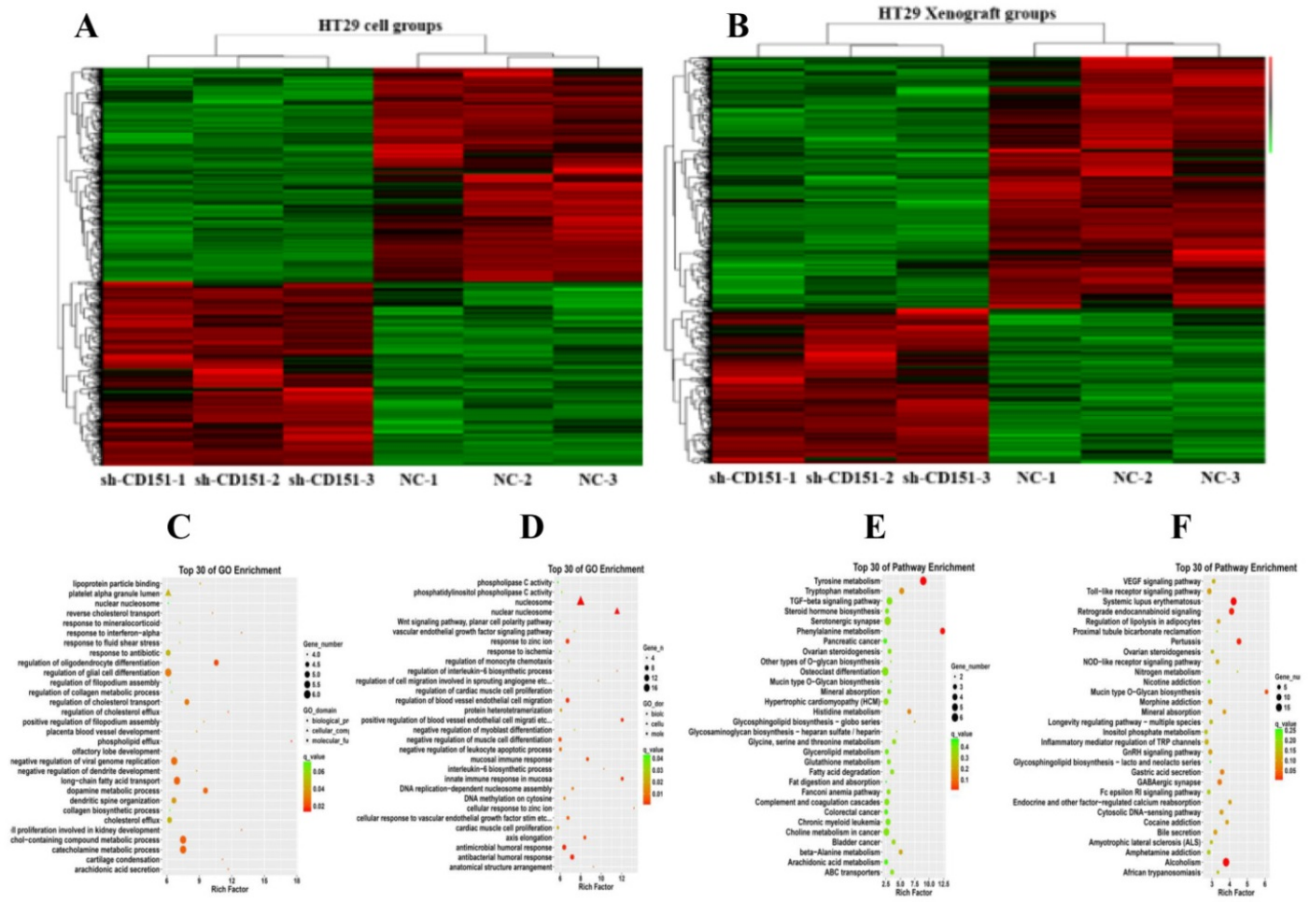
** RNA-seq data for transcriptomic analysis on *in-vitro* cultured CRC cells and *in-vivo* xenograft tumors without and with sh-CD151 transfection in HT29 cells. A and B.** Heat map of up/downregulated unigenes in cultured CRC cells (A) and xenograft tumors (B) with versus without sh-CD151 transfection in HT29 cells. **C and E.** Illustration of top 30 GO- (C) and KEGG pathway- (E) enriched differentially expressed genes (DEGs) in cultured HT29 cells with sh-CD151 transfection. **D and F.** Illustration of top 30 GO- (D) and KEGG pathway- (F) enriched DEGs in xenograft tumors derived from sh-CD151 transfected HT29 cells.

To investigate the molecular mechanisms by which CD151 knockdown regulates tumorigenesis**,** the transcriptomic profiles of HT29 cells and derived colon xenografts in nude mice were examined by RNA-seq. In this study, 724 and 586 significantly differentially expressed genes (SDEGs) were found in cell and xenograft groups, respectively, which included 243 SDEGs found in both cell and xenograft groups. A total of 279 exclusive and 114 common SDEGs were upregulated, while 202 exclusive and 129 common SDEGs were downregulated in HT29 cells treated with sh-CD151 versus control cells. Meanwhile, 117 exclusive and 314 common SDEGs were upregulated, while 226 exclusive and 329 common SDEGs were downregulated in colon xenografts from cells treated with sh-CD151 versus controls. GO and KEGG pathway analysis of DEGs in HT29 cells and xenografts revealed that CD151 was involved in multiple cancer signaling pathways, including the TGFβ, Wnt, Notch, Hedgehog and vascular endothelial growth factor (VEGF) signaling pathways, and regulated signal transduction effectors such as LGR5, bone morphogenetic protein 4 (BMP4) and TGFβ receptor type 1 (TGFβR1) (**Figure 5**).

### Proteins identified by CoIP-MS and validation of LGR5, TGFβ1, and CEACAM6 as binding partners of CD151

**Figure 6:**
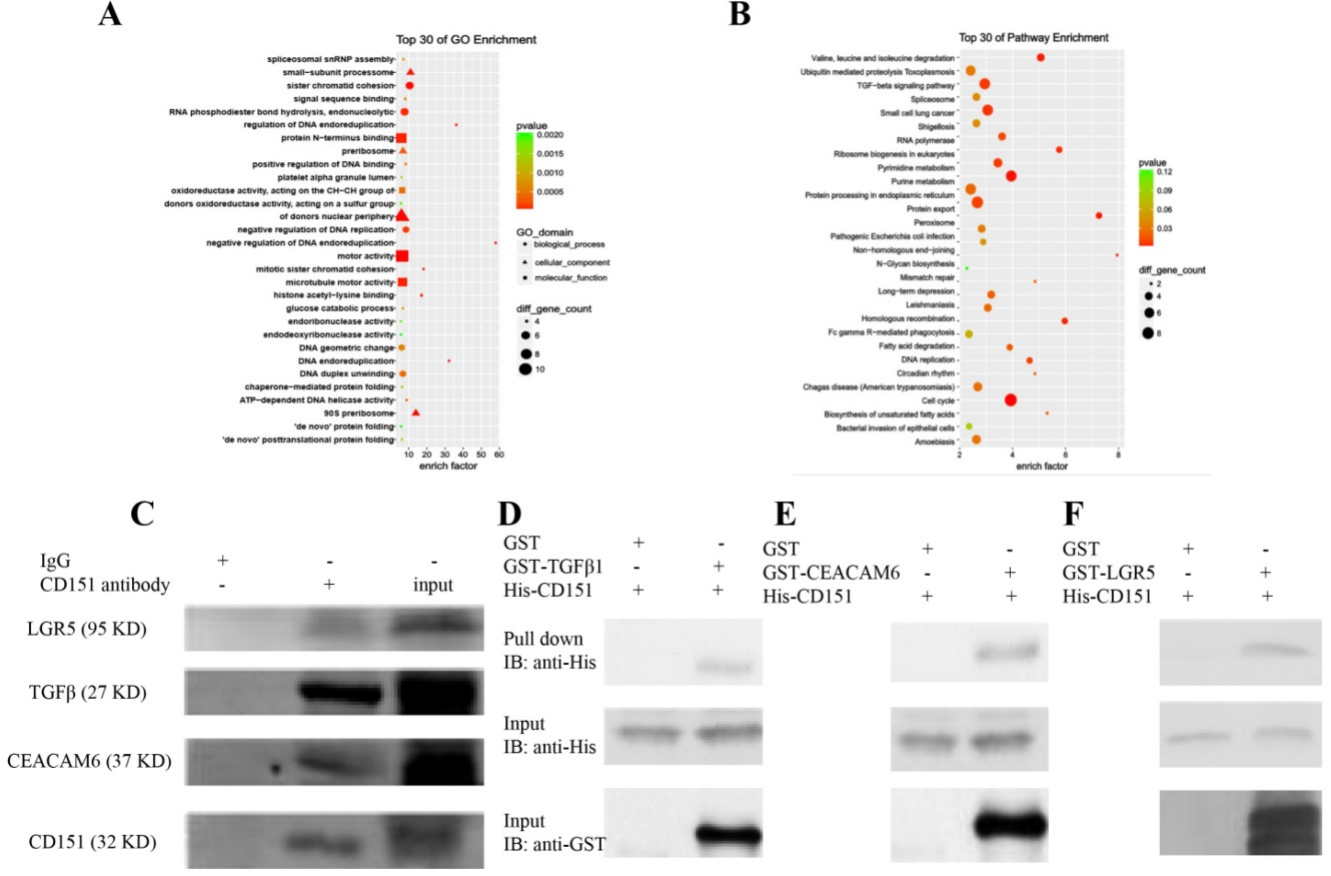
** Proteins were identified by CoIP-MS, with LGR5, TGFβ1 and CEACAM6 validated as binding partners of CD151. A and B**. Illustration of GO (A) and KEGG pathway enrichment (B) of proteins interacting with CD151 as identified by mass spectrometry (MS). **C.** Co-immunoprecipitation (CoIP) showed that LGR5, TGFβ1 and CEACAM6 formed a complex with CD151. **D, E and F.** The direct interactions of GST-tagged TGFβ1 (D), CEACAM6 (E) and LGR5 (F) with His-tagged CD151 were detected by the GST-His pull-down assay.

**Figure 7:**
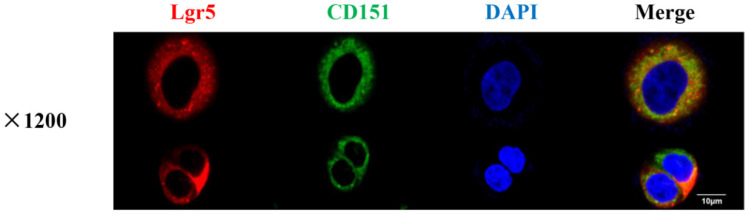
** CD151 co-localizes with LGR5 as demonstrated by immunofluorescence.** Red fluorescence (Lgr5) and green fluorescence (CD151) were shown to overlap in HT29 cells, with cell nuclei counterstained with DAPI in blue (magnification ×1200; Scale bar, 10 µm).

CoIP coupled with MS was performed to further investigate the proteins interacting with CD151. A total of 2268 and 2014 proteins were identified in the experimental and control groups, respectively. Of these, 472 proteins had fold changes greater than two in the experimental group compared with the control group. GO and KEGG pathway analysis indicated that they were involved in multiple signaling pathways, including TGFβ1 signaling, the VEGF/Wnt/Jak-STAT signaling pathway, as well as pathways in the cell cycle, cancer and colorectal cancer (**Figure 6A-C**). GO enrichment analysis showed that specific proteins, including TGFβ1, adenomatous polyposis coli (APC), CTNB1, low-density lipoprotein receptor-related protein (LRP) 5 (LRP5), LRP6, JAK1, ST14, and TP53BP1, were hypothetical CD151-interacting proteins in colon cancer. The CoIP-MS results were consistent with RNA-seq data. To further verify LGR5, CEACAM6, and TGFβ1 as potential proteins interacting with CD151, a pull-down assay was performed, confirming that CD151 could interact with LGR5, CEACAM6 and TGFβ1 *in vitro* (**Figure 6D-F**). Moreover, immunofluorescence indicated that LGR5 co-localized with CD151 in HCT116 cells. As confirmed in Figure 7, red fluorescence (LGR5) and green fluorescence (CD151) were overlapped on HT29 cells (**Figure 7**).

### LGR5, CEACAM6 and Wnt pathways are suppressed by shCD151 in HT29 and HCT116 cells *in vitro* and* in vivo*

**Figure 8:**
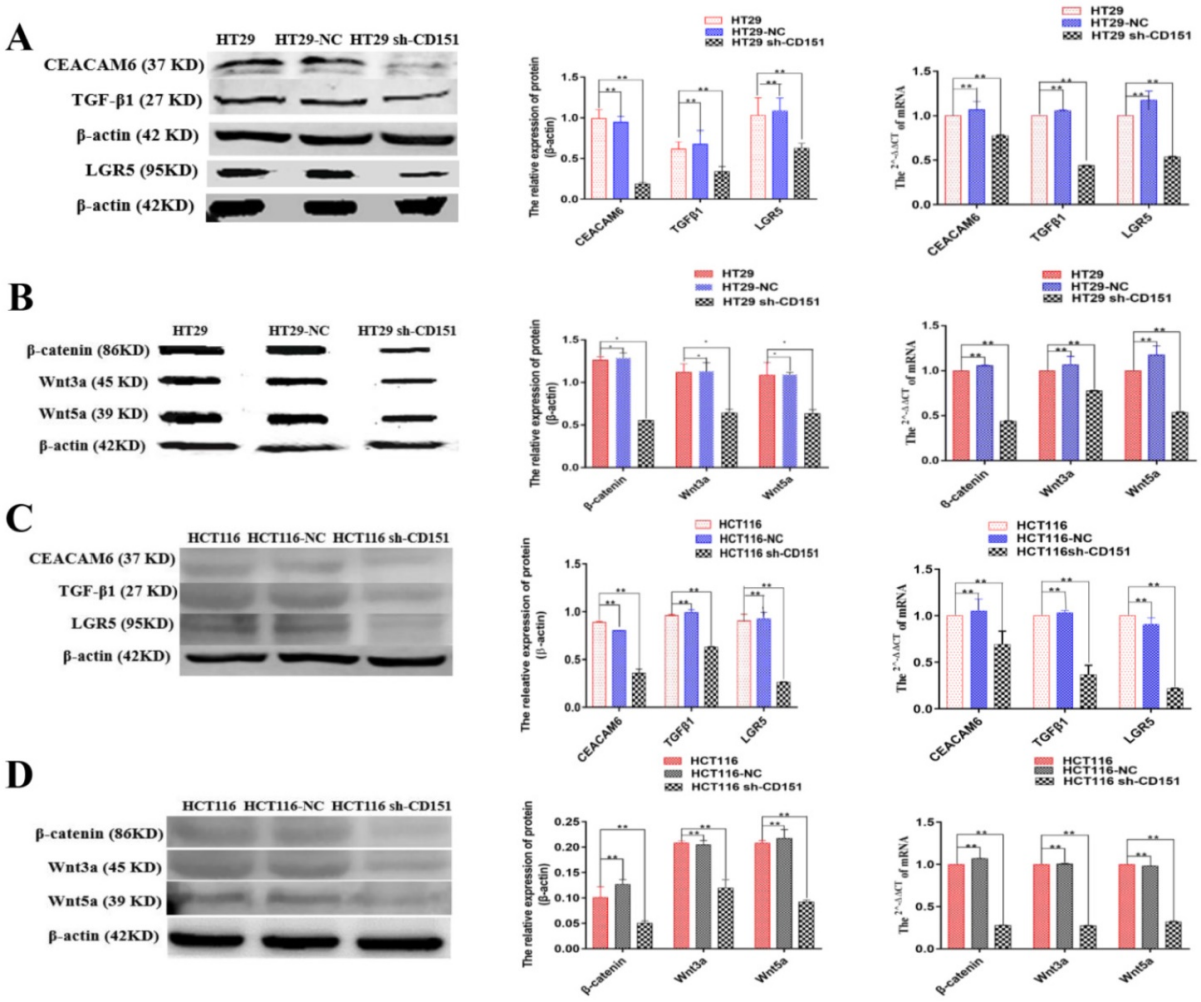
** CEACAM6, TGFβ1, LGR5, β-catenin, Wnt3a and Wnt5a are downregulated by CD151 silencing in HT29 and HCT116 cells. A and B.** Western blot and qRT-PCR showed that CEACAM6, TGFβ1, LGR5 (A), β-catenin, Wnt3a and Wnt5a (B) were downregulated at both protein and mRNA levels by CD151 silencing in HT29 cells, as compared with controls. **C and D.** Western blot and qRT-PCR demonstrated CEACAM6, TGFβ1, LGR5 (C), and β-catenin, Wnt3a and Wnt5a (D) were downregulated at both protein and mRNA levels by CD151 silencing in HCT116 cells, as compared with controls. *P < 0.05, **P < 0.01.

**Figure 9:**
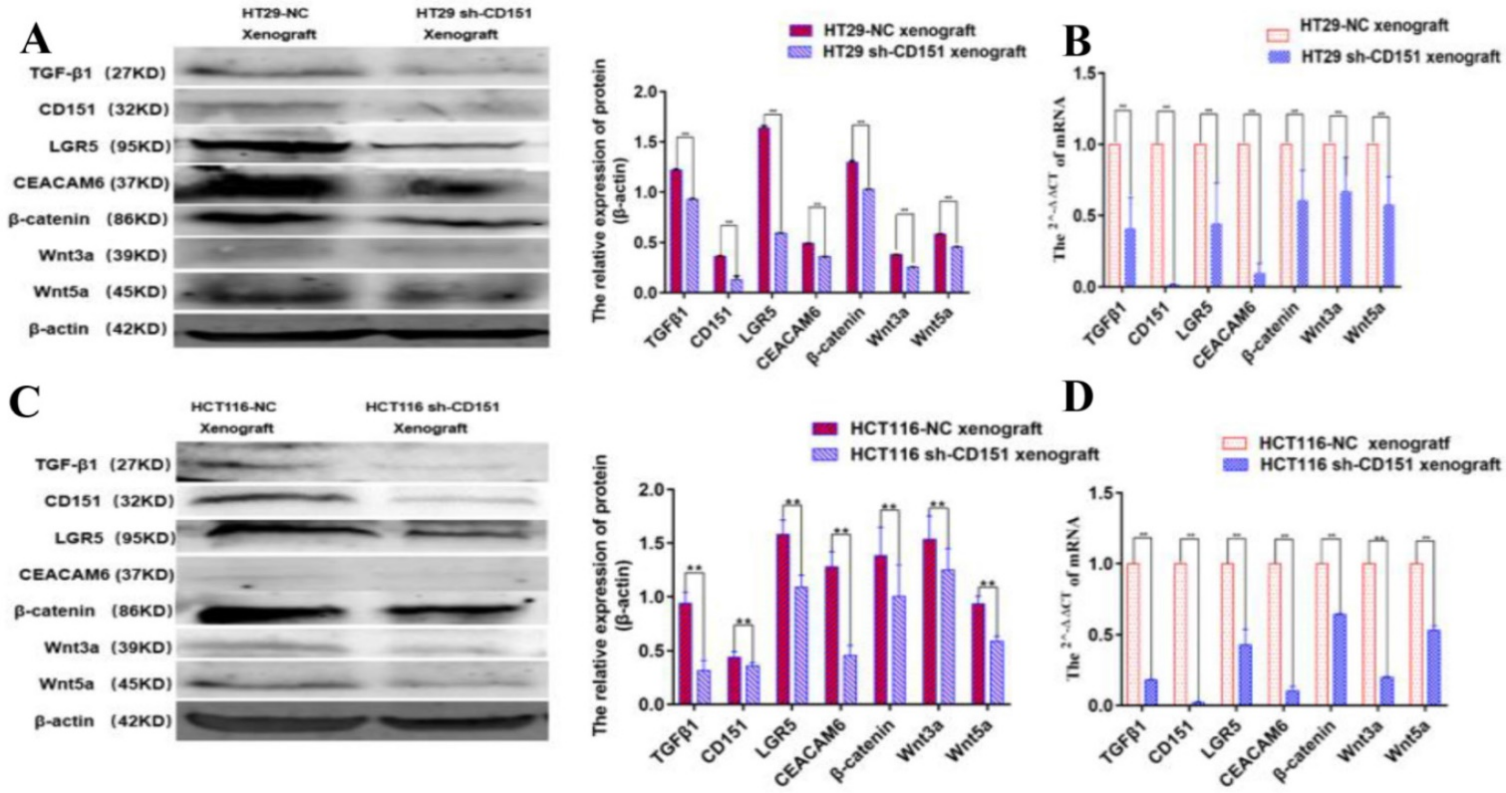
** TGFβ1, CD151, LGR5, CEACAM6, β-catenin, Wnt3a and Wnt5a are downregulated in xenograft tumors derived from CD151-silenced HT29 or HCT116 cell implant. A and B.** Western blot and qRT-PCR showed TGFβ1, CD151, LGR5, CEACAM6, β-catenin, Wnt3a and Wnt5a were downregulated at both protein and mRNA levels in xenograft tumors generated by sh-CD151 transfected HT29 cell implant, as compared with controls (HT29-NC xenograft). **C and D.** Western blot and qRT-PCR showed TGFβ1, CD151, LGR5, CEACAM6, β-catenin, Wnt3a and Wnt5a were downregulated at both protein and mRNA levels in xenograft tumors generated by sh-CD151 transfected HCT116 cell implant, as compared with controls (HCT116-NC xenograft). *P < 0.05, **P < 0.01.

Western blot and qRT-PCR demonstrated significantly decreased expression levels of LGR5, CEACAM6, β-catenin, Wnt3a and Wnt5a in cultured HT29 and HCT116 cells after CD151 silencing at the protein and mRNA levels, respectively (**Figure 8**). Similar findings were obtained in mouse xenografts (**Figure 9**).

### TGFβ1 activates LGR5, CEACAM6 and Wnt signaling after CD151 silencing

**Figure 10:**
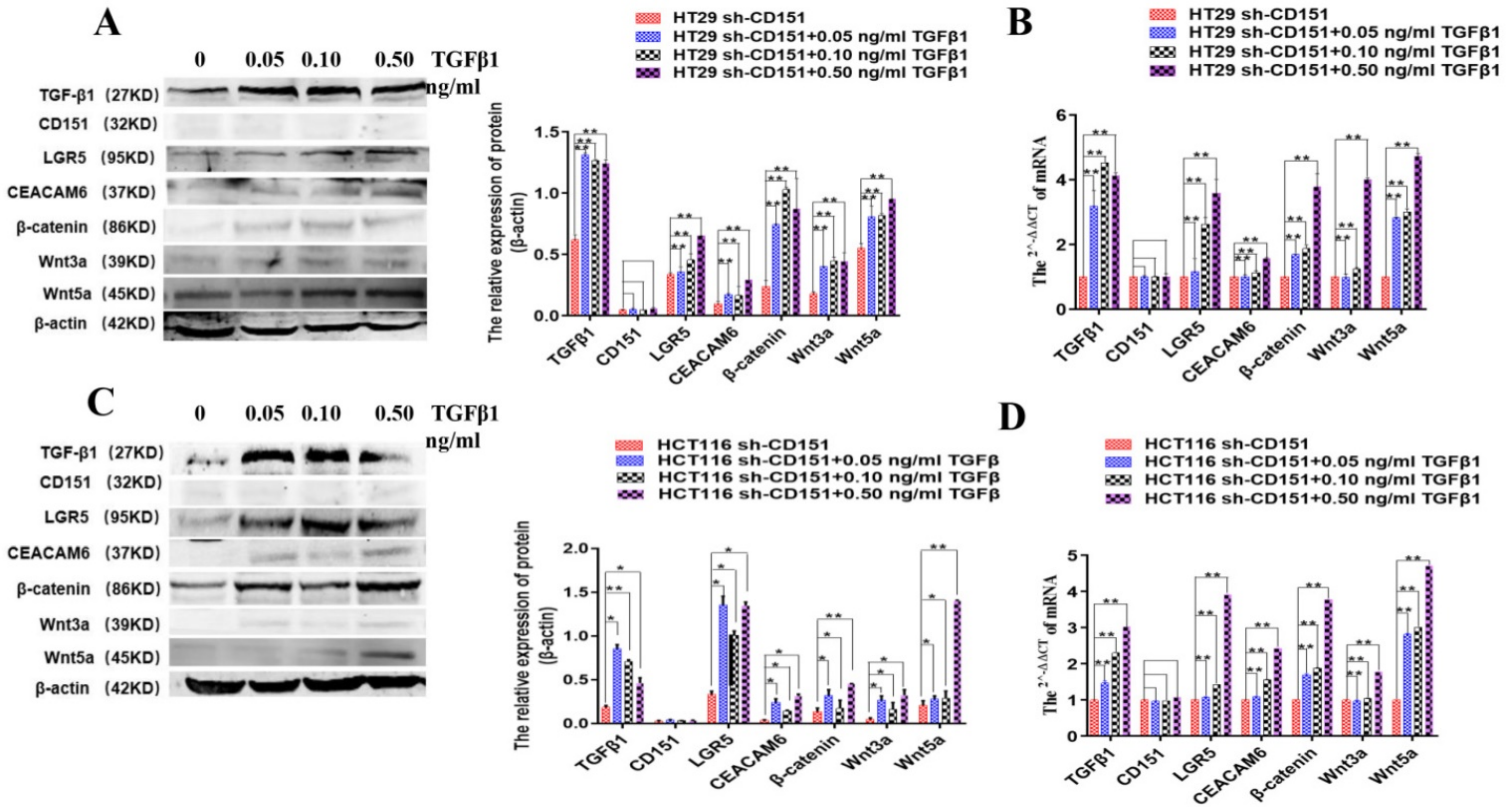
** TGFβ1 additive activates LGR5, CEACAM6 and Wnt signaling after CD151 silencing. A and B.** Western blot and qRT-PCR showed TGFβ1, LGR5, CEACAM6, Wnt3a and Wnt5a were remarkably upregulated at both protein and mRNA levels after treatment with TGFβ1 at 0.05, 0.10 and 0.50 ng/ml, respectively, on sh-CD151 transfected HT29 cells (sh-CD151+TGFβ1 group versus sh-CD151 group). **C and D.** Western blot and qRT-PCR showed TGFβ1, LGR5, CEACAM6, Wnt3a and Wnt5a were remarkably upregulated at both protein and mRNA levels after treatment with TGFβ1 at 0.05, 0.10 and 0.50 ng/ml, respectively, on sh-CD151 transfected HCT116 cells (sh-CD151+TGFβ1 group versus sh-CD151 group). TGFβ1 additive had no impacts on CD151 in the aforementioned groups.

It was previously reported that TGFβ1 upregulation after CD151 silencing could revive malignancy, suggesting that CD151 may promote renal cell carcinoma partially by regulating TGFβ1 amounts and inducing TGFβ1/Smad signaling 16. Therefore, to further investigate the effects of TGFβ1 on LGR5, CEACAM6 and Wnt signaling effectors with CD151 knockdown in this study, sh-CD151 HT29 and HCT116 cells were treated with TGFβ1 (0.05, 0.1 and 0.5 ng/ml) for 48 h. Compared with the sh-CD151 groups, LGR5, CEACAM6, β-catenin, Wnt3a and Wnt5a protein and mRNA levels were starkly increased in the sh-CD151+TGFβ1 groups; Meanwhile, TGFβ1 additive had no impacts on CD151 in the aforementioned groups (**Figure 10**).

## Discussion

The present study comprehensively assessed the role of CD151 in CRC, and demonstrated that CD151 could promote malignancy via multiple pathways, both in cultured cells and in xenograft mouse models. It has been reported that overexpression of CD151 enhances tumor invasion and metastasis, and reduces overall survival in various cancers 29,30. In this study, CD151 was upregulated in CRC tissue samples, and a negative correlation was found between CD151 expression and the survival rate of CRC patients, corroborating the above conclusion. In addition, CD151 silencing was shown to suppress cell migration and invasion in cultured CRC cells *in vitro*, and reduce tumor growth in mouse xenograft models. RNA-seq further demonstrated that CD151 modulated several molecular processes responsible for CRC progression, including TGFβR1 binding, cell motility, activation of calcium channel inhibitors, biological response to stress, immune system activation, signal transducer activities, and et al.

Impacts of CD151 silencing were observed in both cultured cells and mouse xenografts. Among the SDEGs common in both cells and xenograft groups, 114 were upregulated and 129 were downregulated, among which TGFβ1, CEACAM6 and LGR5 were found to be downregulated along with CD151. These findings suggest that TGFβ1, CEACAM6 and LGR5 might be correlated with CD151 in CRC. These data were consistent with previous studies reporting that CD151 promotes cell migration, invasion and metastasis via TGFβ1/Smad signaling 19,31. Here, high TGFβ1 expression was responsible for the aggressiveness of CRC both *in vitro* and *in vivo*. Among the well-known TGFβ family members (TGFβ1, TGFβ2, and TGFβ3), CRC patients are more likely to show recurrence bearing high levels of TGFβ1, which regulates a number of biological processes such as CRC cell proliferation, differentiation, apoptosis, extracellular matrix adhesion and immune response 16,26,27,32.

This study updated our knowledge on CD151 function in cancer progression. Notably, LGR5 co-downregulation with CD151 was identified in xenograft models by RNA-seq, and then validated by Western blot and qRT-PCR in cultured CRC cells and xenograft models. CoIP followed by MS was utilized to confirm that TGFβ1, LRP5, LRP6, CEACAM6 and LGR5 may act as specific CD151 binding partners *in vivo*. Previous reports revealed that LRP5/6 could function as co-receptors in the Wnt signaling pathway by binding to LGR5 33. Here we provided the first direct evidence showing that the TGFβ1-CEACAM6-LGR5 complex interacts with CD151 via RNA-seq, Co-IP-MS, pull-down assay and immunofluorescence. These results suggest that TGFβ1, CEACAM6, and LGR5 act as CD151 binding proteins in CRC. Consistently, we demonstrated that CD151 knockdown in CRC could downregulate TGFβ1, CEACAM6 and LGR5 expression levels. On the other hand, sh-CD151 transfected cells treated with TGFβ1 additive could exhibit restored expression of CEACAM6, LGR5, Wnt3a, Wnt5a and β-catenin. These findings demonstrated that suppression of CEACAM6, LGR5 and Wnt signaling by CD151 silencing may revive via TGFβ1 upregulation, confirming that CD151 promotes CRC progression by a crosstalk involving CEACAM6, LGR5 and Wnt signaling via TGFβ1. Consistently, previous studies reported that regulatory T-cells (Tregs) promote LGR5 upregulation in gastric cancer cells through TGFβ1 signaling, with a likely involvement of Wnt signaling; Meanwhile, high LGR5 levels upregulated by TGFβ1 was thought to deteriorate gastric cancer patient prognosis 34. However, whether Tregs also affect CD151 expression levels deserves further attention.

Although the current study provided novel insights into the mechanism by which CD151 induces malignancy, further investigation is required to explore the exact mechanism underlying the interaction and regulation between CD151 and the aforementioned proteins. Additional molecules screened in RNA-seq and CoIP also need to be further assessed, and future efforts will be directed towards that end.

## Conclusion

Taken together, this study demonstrated that CD151 plays a pivotal role in CRC progression, during which process CD151 functions through TGFβ1 to regulate CEACAM6, LGR5 and Wnt signaling, thus offering a comprehensive view of CD151's roles in colorectal carcinogenesis. Our findings provide an insight into the CD151-involved signaling network in oncogenesis of CRC, which could be utilized to design novel targeted therapies against CD151-based signaling during the treatment for CRC.

## Figures and Tables

**Table 1:** Primers used in this study

Gene	Forward (5'-3')	Reverse (5'-3')
CD151	ATTGCCTGTGTGCAGGTCTT	TCAGTAGTTGGGTGCAGCAG
LGR5	GCATTTGTAGGCAACCCTTC	TTGTGAGGCACCATTCAGAG
CEACAM6	GTCCTGCTCACAGCCTCACTTC	ACTGTTGCCATCCACTCTTTCG
TGFβ1	CAGCAACAATTCCTGGCGATAC	GCTAAGGCGAAAGCCCTCAA
Wnt3a	CCATTTGCGGCTGTGACT	GCCTCGTTGTTGTGCTTGT
Wnt5a	ATTCTTTGGTGGTCGCTAGG	CTGTCCTTGAGAAAGTCCTG
β-catenin	TTGAAAATCCAGCGTGGACA	TCGAGTCATTGCATACTGTC
β-actin	TGACGTGGACATCCGCAAAG	CTGGAAGGTGGACAGCGAGG

**Table 2:** GST pull-down primers

	GST pull-down primer
CD151-F	ATGGGTCGCGGATCCGAATTCATGGGTGAGTTCAACGAG
CD151-R	GTGGTGGTGGTGGTGCTCGAGTCAGTAGTGCTCCAGCTTG
LGR5-F	GATCTGGTTCCGCGTGGATCCATGGACACCTCCCGGCTC
LGR5-R	GTCACGATGCGGCCGCTCGAGTTAGAGACATGGGACAAATGC
CEACAM-F	GATCTGGTTCCGCGTGGATCCATGGGTCCACCAAGTGCC
CEACAM-R	GTCACGATGCGGCCGCTCGAGTTAAATCAGCGCCACACG
TGFβ1-F	GATCTGGTTCCGCGTGGATCCATGCCACCGAGCGGTCTG
TGFβ1-R	GTCACGATGCGGCCGCTCGAGTTAGCTACACTTGCAGCTG

**Table 3:** Tissue microarray staining results and clinicopathological characteristics in colon cancer patients

Variables	Negative (%)	Positive (%)	Total	*P* value
**All cases**	20 (25%)	60 (75%)	80	
**Age**				
≤55years	4 (16.7%)	20 (83.3%)	24	0.26
>55 years	16 (28.6%)	40 (71.4%)	56	
**Gender**				
Male	10 (28.6%)	25(71.4%)	35	0.515
Female	10 (22.2%)	35 (77.8%)	45	
**Tumor size**				
≤ 5 cm	11 (28.9%)	27 (70.1%)	38	0.438
>5 cm	9 (21.4%)	33 (78.6%)	42	
**pT status**				
pT_2_	4 (50%)	4 (50%)	8	0.102
pT_3_pT_4_	16 (22.2%)	56 (77.8%)	72	
**pN status**				
pN_0_	13 (35.1%)	24 (64.9%)	37	0.198
pN_1_ pN_2_	17 (21.2%)	26 (78.8%)	43	
**pM status**				
pM_0_	9 (60%)	6 (40%)	15	0.001
pM_1_	11 (16.9%)	54 (83.1%)	65	
**TNM stage**				
I-II	7 (18.9%)	30 (81.1%)	37	0.244
III-IV	13 (30.2%)	30 (69.8%)	43	

A total of 80 colon cancer patients were assessed by immunohistochemistry for CD151. *Two sided Fisher's exact test.
