# CD151 promotes Colorectal Cancer progression by a crosstalk involving CEACAM6, LGR5 and Wnt signaling via TGFβ1: Erratum

**DOI:** 10.7150/ijbs.84867

**Published:** 2023-06-21

**Authors:** Tao Yang, Huibing Wang, Meng Li, Linqi Yang, Yu Han, Chao Liu, Baowen Zhang, Mingfa Wu, Gang Wang, Zhenya Zhang, Wenqi Zhang, Jianming Huang, Huaxing Zhang, Ting Cao, Pingping Chen, Wei Zhang

**Affiliations:** 1 Center for Medical Research and Innovation, Shanghai Pudong Hospital, Fudan University Pudong Medical Center, Shanghai, 201399, China.; 2 Department of Pharmacology, Hebei University of Chinese Medicine, Shijiazhuang, Hebei, 050200, China.; 3 Department of Pharmacy, Children's Hospital of Hebei Province, Shijiazhuang, Hebei, 050000, China.; 4 Department of Laboratory Animal Science, Hebei Key Lab of Hebei Laboratory Animal Science, Hebei Medical University, Shijiazhuang, Hebei, 050017, China.; 5 Hebei Collaboration Innovation Center for Cell Signaling, Key Laboratory of Molecular and Cellular Biology of Ministry of Education, Hebei Key Laboratory of Moleculor and Cellular Biology, College of Life Sciences, Hebei Normal University, Shijiazhuang, Hebei, 050024, China.; 6 Department of Gastrointestinal Surgery, Dingzhou City People's Hospital, Dingzhou, Hebei, 073000, China.; 7 Department of Third General Surgery, Cangzhou City People's Hospital, Cangzhou, Hebei, 061000, China.; 8 Department of Second General Surgery, Hebei Medical University Fourth hospital, Shijiazhuang, Hebei, 050011, China.; 9 College of Basic Medicine, Hebei Medical University, Shijiazhuang, Hebei, 500017, China.; 10 School of Basic Medical Sciences, Hebei Medical University, Shijiazhuang 050017, Hebei, China.

After a periodic review of the publication from our lab, we found an error in the Western blotting images of Figure 8 D Wnt3a, which occurs during our image arrangement at the time of manuscript submission. The other elements of the Figure 8 D remain the same, and the interpretation of the results remains unchanged. The corrected figure is presented below. All authors have agreed to the erratum and we apologize for any inconvenience caused by the negligence in our work.

## Figures and Tables

**Figure 8:**
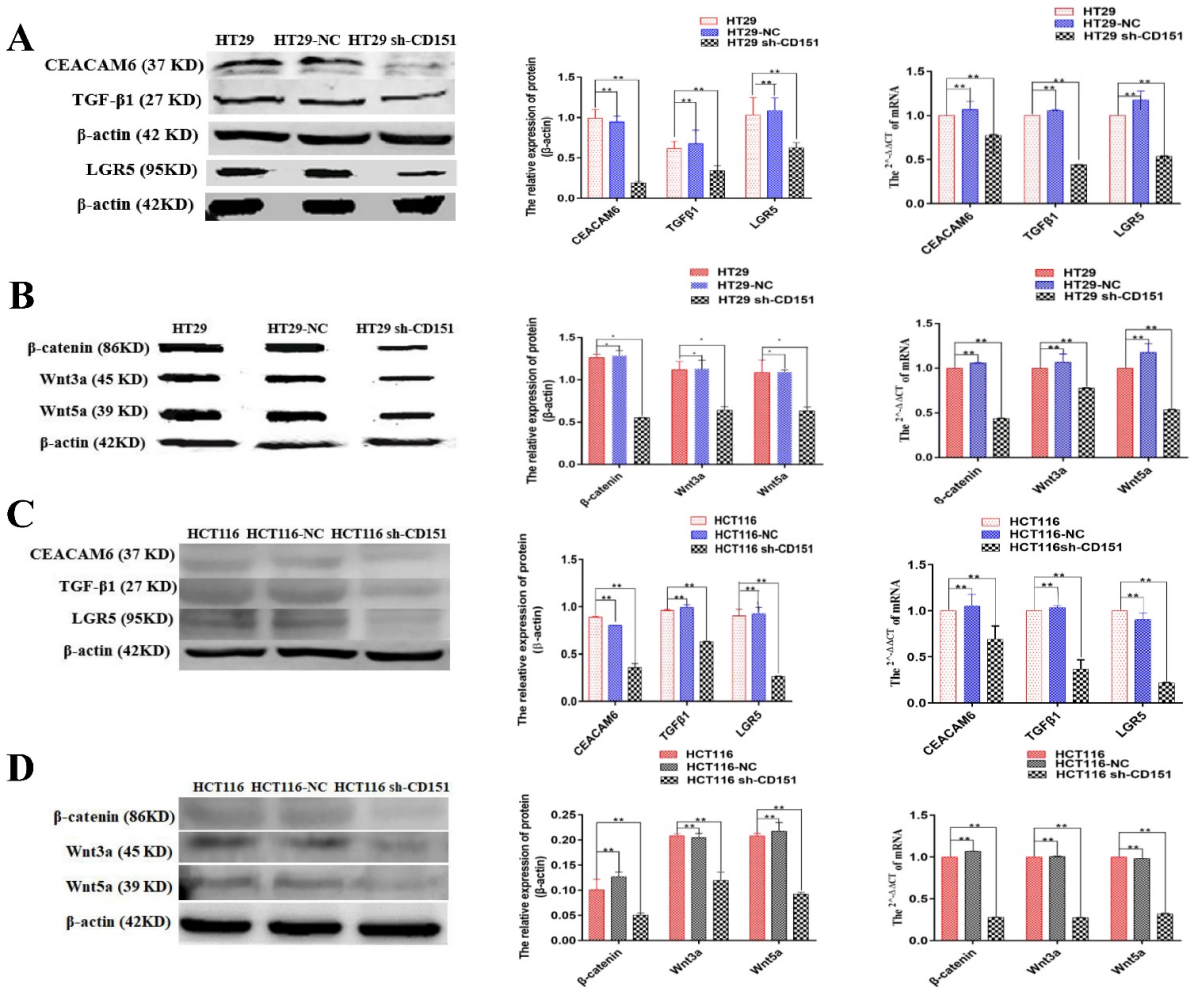
Correct image.

